# Gintonin Stimulates Glucose Uptake in Myocytes: Involvement of Calcium and Extracellular Signal-Regulated Kinase Signaling

**DOI:** 10.3390/biom14101316

**Published:** 2024-10-17

**Authors:** Rami Lee, Kyung-Jong Won, Ji-Hun Kim, Byung-Hwan Lee, Sung-Hee Hwang, Seung-Yeol Nah

**Affiliations:** 1 Ginsentology Research Laboratory and Department of Physiology, College of Veterinary Medicine, Konkuk University, Seoul 05029, Republic of Korea; rmlee12@konkuk.ac.kr (R.L.); bioskjh@konkuk.ac.kr (J.-H.K.); 2 Department of Physiology and Premedical Science, College of Medicine, Konkuk University, Chungju 27478, Republic of Korea; kjwon@kku.ac.kr; 3 Jeju Self-Governing Provincial Veterinary Research Institute, Jeju 63344, Republic of Korea; mischief76@korea.kr; 4 Department of Pharmaceutical Engineering, College of Health Sciences, Sangji University, Wonju 26339, Republic of Korea

**Keywords:** gintonin, skeletal muscle, C2C12, glucose uptake, calcium, ATP, GLUT4, sarcopenia

## Abstract

Ginseng has anti-hyperglycemic effects. Gintonin, a glycolipoprotein derived from ginseng, also stimulates insulin release from pancreatic beta cells. However, the role of gintonin in glucose metabolism within skeletal muscle is unknown. Here, we showed the effect of gintonin on glucose uptake, glycogen content, glucose transporter (GLUT) 4 expression, and adenosine triphosphate (ATP) content in C2C12 myotubes. Gintonin (3–30 μg/mL) dose-dependently stimulated glucose uptake in myotubes. The expression of GLUT4 on the cell membrane was increased by gintonin treatment. Treatment with 1–3 μg/mL of gintonin increased glycogen content in myotubes, but the content was decreased at 30 μg/mL of gintonin. The ATP content in myotubes increased following treatment with 10–100 μg/mL gintonin. Gintonin transiently elevated intracellular calcium concentrations and increased the phosphorylation of extracellular signal-regulated kinase (ERK). Gintonin-induced transient calcium increases were inhibited by treatment with the lysophosphatidic acid receptor inhibitor Ki16425, the phospholipase C inhibitor U73122, and the inositol 1,4,5-trisphosphate receptor antagonist 2-aminoethoxydiphenyl borate. Gintonin-stimulated glucose uptake was decreased by treatment with U73122, the intracellular calcium chelator 1,2-bis(o-aminophenoxy)ethane-N,N,N′,N′-tetraacetic acid tetra(acetoxymethyl) ester, and the ERK inhibitor PD98059. These results show that gintonin plays a role in glucose metabolism by increasing glucose uptake through transient calcium increases and ERK signaling pathways. Thus, gintonin may be beneficial for glucose metabolism control.

## 1. Introduction

Sarcopenia, characterized by the progressive loss of muscle mass and strength with aging, has emerged as a critical health issue impacting the elderly population globally [1,2]. This condition not only diminishes physical function and quality of life, but also has been increasingly linked to several metabolic health concerns, particularly type 2 diabetes mellitus (T2DM) [2,3,4]. T2DM is characterized by insufficient insulin secretion or insulin resistance [5]. Recent studies have reported a complex interplay between sarcopenia and T2DM, showing that declines in skeletal muscle mass and function may worsen glucose dysregulation and contribute to the development of insulin resistance, and that T2DM may increase the risk of sarcopenia [2,3]. Glucose levels are kept stable by complex homeostatic mechanisms. Glucose homeostasis can be controlled by glucose output, glucose release, or glucose uptake in different tissues, such as the liver, pancreas, adipose, and skeletal muscle [6,7]. Skeletal muscle plays an important role in glucose metabolism because it is the primary site of insulin-stimulated glucose uptake [8], thereby contributing to the regulation of blood glucose levels and the preservation of stable glucose balance [6]. In this context, improving impaired glucose homeostasis in skeletal muscle may be a therapeutic strategy to overcome glucose dysregulation in disorders such as sarcopenia and T2DM.

Glucose uptake in skeletal muscle serves as an important regulator in maintaining normal glucose homeostasis [7]. Glucose uptake-related processes in skeletal muscle cells include various molecules, including calcium, adenosine triphosphate (ATP), and glucose transporter (GLUT) 4 [9,10,11]. Calcium, a ubiquitous second messenger, orchestrates a myriad of cellular responses, including glucose uptake [11]. Transient increases in intracellular calcium concentrations in skeletal muscle cells are associated with GLUT4 translocation and glucose transport, and calcium can also stimulate glucose transport through various pathways in skeletal muscle, which may facilitate glucose uptake [12]. ATP, a source of cellular energy, is intimately linked to glucose uptake processes [9]. ATP-deficient conditions increase surface GLUT levels and glucose transport in skeletal muscle [13]. GLUT4 is the main glucose transporter protein expressed in skeletal muscle and is essential for the uptake of glucose in skeletal muscle triggered by insulin [13]. Therefore, any dysregulation in calcium, adenosine triphosphate (ATP), or GLUT4 in skeletal muscle may compromise glucose homeostasis by interfering with glucose uptake.

Ginseng has been reported to have beneficial effects on insulin resistance and sarcopenia [14]. Gintonin, a bioactive component extracted from Korean Ginseng, has also been reported to have health benefits [15]. Recent research has shown that gintonin is a lysophosphatidic acid (LPA) receptor ligand and stimulates the proliferative and migratory activities in various cell types, including endothelial cells and keratinocytes [15,16,17]. Gintonin has also been shown to induce transient elevations in intracellular calcium concentrations in several cell types expressing LPA receptors via LPA receptor activation [15,16]. The stimulation of LPA receptors by LPA regulates glucose metabolism by promoting glucose uptake by muscle cells [18]. In addition, gintonin can increase insulin secretion from pancreatic beta cells, indicating potential anti-diabetic activity [19]. Gintonin also has anti-atrophy effects on skeletal muscle [20]. In this context, gintonin may be an interesting exploration target for glucose uptake research on recovering muscle function in patients with T2DM and sarcopenia. Therefore, unraveling the complex interplay between gintonin and glucose uptake-related responses in skeletal muscle may provide a mechanistic understanding of the potential therapeutic impact of gintonin in alleviating muscle dysfunction related to glucose homeostasis.

Although studies have demonstrated diverse beneficial bioactivities of gintonin, the influence of gintonin on the responses related to glucose uptake in skeletal muscle is unknown. In the present study, we set out to explore the modulatory effects of gintonin on skeletal muscle glucose uptake, with a specific focus on its interactions with calcium signaling, ATP levels, and GLUT4 dynamics in C2C12 cells. Through these explorations, we also aimed to suggest the potential use of gintonin as a candidate for the development of new therapeutics for improving abnormal glucose homeostasis in skeletal muscle.

## 2. Materials and Methods

### 2.1. Materials

Gintonin originated from *Panax ginseng*, as noted in an earlier study [21]. LPA (C18:1) was obtained from Avanti Polar Lipids in Alabaster, AL, USA. Anti-phospho-ERK1/2 (p-ERK) and anti-ERK antibodies were acquired from Cell Signaling Technology, Inc. (Danvers, MA, USA). Anti-GLUT4 and anti-Na^+^/K^+^-ATPase α antibodies were sourced from Santa Cruz Biotechnology (Dallas, TX, USA). A mouse monoclonal β–actin conjugated with horseradish peroxidase (HRP) was obtained from Abcam (Cambridge, MA, USA). Goat anti-rabbit IgG antibody was sourced from GeneTex (Irvine, CA, USA). Ki16425 and the glycogen assay kit were supplied by Cayman Chemical Co. (Ann Arbor, MI, USA). Materials for cell culture were obtained from Thermo Fisher Scientific (Seoul, Republic of Korea). The ATP assay kit, PhoStop, and additional materials were soured from Merck Korea (Seoul, Republic of Korea).

### 2.2. Cell Culture

The C2C12 mouse myoblast cell line was provided by Prof. J.-Y. Imm (Kukmin University, Seoul, Republic of Korea). C2C12 cells were cultured in Dulbecco’s modified Eagle’s medium (DMEM) with an addition of 10% fetal bovine serum (FBS), along with penicillin at 100 units/mL and streptomycin at 100 μg/mL. For differentiation to myotubes, cells were exposed to DMEM supplemented with 2% horse serum for a duration of five days [22].

### 2.3. Cell Viability Assay

Cell viability was analyzed through a water-soluble tetrazolium formazan (WST) assay utilizing EZ-Cytox (Dogen, Seoul, Republic of Korea), following the instructions provided by the manufacturer. The differentiated myotubes were treated with gintonin or LPA in 96-well plates for 24 h. Afterward, the culture medium changed to a serum-free medium devoid of phenol red, and the myotubes were exposed to EZ-Cytox solution for 2 h. The absorbance was then recorded at 450 nm with a plate reader, the Spectra Max 190 (Molecular Devices, Sunnyvale, CA, USA).

### 2.4. Measurement of [Ca^2+^]_i_ in Cell Suspensions

The C2C12 myotubes were subjected to trypsin/EDTA treatment to facilitate detachment. Afterwards, they were washed with HEPES-buffered saline solution at pH 7.4, and then incubated with fura-2-acetoxymethyl ester (AM) (2.5 μM). Cells loaded with Fura-2-AM were treated with gintonin (GT, 0.3–30 μg/mL), and intracellular free calcium levels were evaluated through dual excitation spectrofluorometry (excitation: 340 nm and 380 nm, emission: 510 nm) using a spectrofluorophotometer (RF-5301PC, Shimazu Scientific Korea, Seoul, Republic of Korea), as previously described [16]. In order to examine the effect of inhibitors, Fura-2-AM-loaded C2C12 myotubes were pretreated with PTX (100 ng/mL), Ki16425 (10 μM), U73122 (5 μM), or 2-APB (100 μM) for 5 min and then treated with gintonin (GT, 1 μg/mL).

### 2.5. ATP Assay

The C2C12 myotubes were treated with gintonin at the specified concentrations for the indicated times in 96-well plates. The medium was removed from each well, and dilution buffer and cell lysis reagent were added. The ATP content of the cell lysates was measured using an ATP assay kit, in line with instructions provided by the manufacturer (Merck Korea, Seoul, Republic of Korea). Luciferase reagent was added to each well using an automated injector, and the luminescence intensity was detected using a Veritas microplate luminometer (Turner Biosystems, Sunnyvale, CA, USA). The ATP concentration of each well was calculated from a log–log plot of the standard curve.

### 2.6. Glucose Uptake Assay

The C2C12 myotubes were exposed to gintonin at the specified concentrations, and 100 μM of 2-deoxy-2-[(7-nitro-2,1,3-benzoxadiazol-4-yl)amino]-D-glucose (2-NBDG; Merck Korea, Seoul, Republic of Korea) was added to glucose-free DMEM for 24 h. Then, the cells were washed using phosphate-buffered saline (PBS) chilled on ice. The fluorescence intensity of 2-NBDG in cells was measured using a microplate fluorescence reader (SpectraMax Gemini EM, Molecular Devices, Sunnyvale, CA, USA; excitation: 485 nm, emission: 535 nm).

### 2.7. Glycogen Content Assay

C2C12 myotubes were incubated in serum-free DMEM for 2.5 h. The myotubes were then treated with gintonin at the indicated concentrations or insulin for 120 min. Then, the myotubes were washed with PBS twice and lysed in assay buffer using a sonicator (Vibra Cell, Sonics, Newtown, CT, USA). The cellular glycogen content was measured with a glycogen assay kit, in line with instructions provided by the manufacturer (Cayman Chemical).

### 2.8. Immunoblotting

The levels of phosphorylated ERK in cell lysates were analyzed. The cells were subjected to lysis using a modified radioimmunoprecipitation assay buffer supplemented with inhibitors that target both proteases and phosphatases. Proteins were separated through sodium dodecyl sulfate–polyacrylamide gel electrophoresis and then transferred onto membranes made of polyvinylidene fluoride. The membranes were incubated with a rabbit antibody targeting phospho-ERK at a dilution of 1:1000 and goat anti-rabbit IgG antibody coupled to HRP. The membranes were stripped and re-evaluated using a rabbit anti-ERK antibody (1:1000) and goat anti-rabbit IgG antibody coupled to HRP. The development of the blots involved the use of Clarity Western ECL Substrate from Bio-Rad (Hercules, CA, USA), and images were obtained with the iBright CL1000 imaging system from Thermo Fisher Scientific.

### 2.9. GLUT4 Expression Assay

The myotubes were treated with gintonin or insulin for the specified durations, followed by a wash with PBS and incubation with sulfo-NHS-SS-biotin for cell surface biotinylation. The cells were lysed with lysis buffer, and the labeled cell membrane fraction was isolated using the EZ-link Sulfo-NHS-LC biotinylation kit from Thermo Fisher Scientific (Rockford, IL, USA). The cell membrane fraction and total cell lysate were also used to measure GLUT4 expression by immunoblotting using a mouse anti-GLUT4 monoclonal antibody (Santa Cruz Biotechnology) and a goat anti-mouse IgG antibody conjugated to HRP (Santa Cruz Biotechnology). HRP-conjugated mouse anti-β-actin monoclonal antibody (1:30,000) was used as a loading control for the cell lysates. A mouse anti-Na^+^/K^+^ ATPase α1 monoclonal antibody from Santa Cruz Biotechnology was employed as the loading control for the cell membranes.

### 2.10. Statistical Analysis

The data are shown as the mean ± standard error of the mean (SEM). To assess the significance of the differences between group pairs, the Student’s *t*-test was used. For multiple comparisons, a one-way analysis of variance (ANOVA) was carried out, followed by Dunnett’s post hoc test, utilizing GraphPad Prism (version 6.0; GraphPad Software, Inc., La Jolla, CA, USA). A *p*-value below 0.05 was regarded as significantly different.

## 3. Results

### 3.1. Intracellular Calcium Transient by Gintonin in C2C12 Myotubes

**Figure 1:**
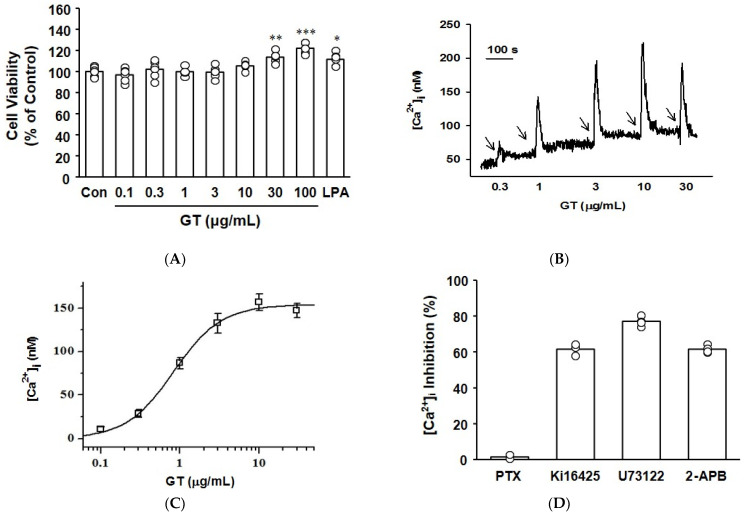
Effect of gintonin on the cell viability of myotubes and transient intracellular calcium increases. (**A**) Cell viability. C2C12 myotubes were treated with gintonin (GT, 0.1–100 μg/mL) or lysophosphatidic acid (10 μM) for 24 h. Then, WST assay was performed. All data are shown as the mean ± SEM (*n* = 6). (**B**,**C**) Transient intracellular calcium increases. Fura-2-AM-incorporated C2C12 myotubes were treated with gintonin (GT, 0.3–30 μg/mL), and intracellular calcium levels were measured by spectrofluorophotometry and calculated. Each arrow in panel (**B**) represents time points of treatment with gintonin at indicated concentrations. The horizontal length of the upper scale bar corresponds to 100 s (100 s). (**D**) Inhibitory effects of inhibitors on GT-induced intracellular calcium increase. Fura-2-AM-loaded C2C12 myotubes were pretreated with PTX (100 ng/mL), Ki16425 (10 μM), U73122 (5 μM), or 2-APB (100 μM) for 5 min and then treated with gintonin (GT, 1 μg/mL). All data are shown as the mean ± SEM (*n* = 3–5); * *p* < 0.05; ** *p* < 0.01; *** *p* < 0.001 vs. untreated control cells (Con). PTX, pertussis toxin; 2-APB, the inositol 1,4,5-trisphosphate receptor antagonist 2-aminoethoxydiphenyl borate.

Gintonin was not cytotoxic to C2C12 myotubes at concentrations ranging from 0.1 to 100 μg/mL (Figure 1A). At 30–100 μg/mL, gintonin increased myotube viability. Gintonin was previously reported to induce transient increases in calcium in several different cell lines via the LPA receptor. Here, Fura-2-AM-loaded C2C12 myotubes were treated with gintonin to evaluate the effect of gintonin on intracellular transient calcium. Intracellular calcium concentrations [Ca^2+^]_i_ were measured by spectrofluorophotometry [16]. Gintonin (0.3–30 μg/mL) treatment induced transient increases in intracellular calcium concentrations in a dose-dependent manner (Figure 1B,C). These transient increases were significantly reduced by the LPA1/3 receptor inhibitor Ki16425, the phospholipase C (PLC) inhibitor U73122, and the inositol 1,4,5-trisphosphate (IP_3_) receptor antagonist 2-aminoethoxydiphenyl borate (2-APB), but not by pretreatment with the G_i_ inhibitor pertussis toxin (Figure 1D). These results indicate that gintonin may induce intracellular calcium transient by activating the LPA receptor, PLC, and the IP_3_ receptor signaling pathway.

### 3.2. Gintonin-Induced Glucose Uptake in C2C12 Myotubes

**Figure 2:**
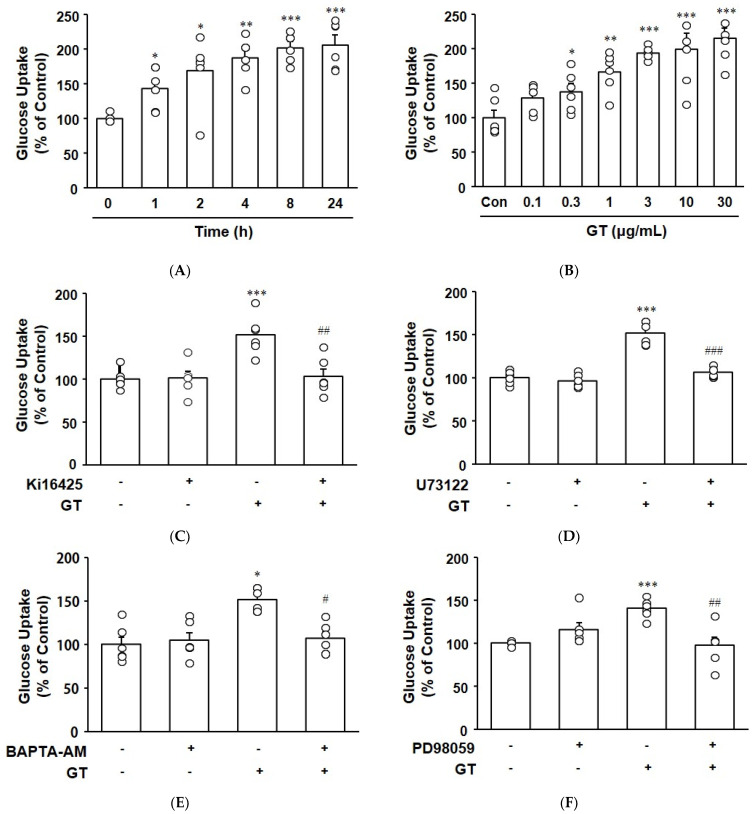
Effect of gintonin on glucose uptake in C2C12 myotubes. (**A**) C2C12 myotubes were treated with gintonin (GT, 3 μg/mL) and 2-NBDG (100 μM) for 0–24 h. (**B**) The myotubes were treated with gintonin (0.1–30 μg/mL) for 24 h. (**C**–**F**) The myotubes were treated with gintonin (GT, 3 μg/mL) and 2-NBDG (100 μM) for 24 h, with or without the addition of inhibitors (Ki16425, 10 μM; U73122, 5 μM; BAPTA-AM, 50 μM; PD98059, 10 μM). Then, 2-NBDG uptake was measured using spectrofluorophotometry. All data are shown as the mean ± SEM. (*n* = 6); * *p* < 0.05; ** *p* < 0.01; *** *p* < 0.001 vs. time 0 or untreated control cells (Con). ^#^ *p* < 0.05; ^##^ *p* < 0.01; ^###^ *p* < 0.001 vs. GT alone. BAPTA-AM, 1,2-bis(o-aminophenoxy)ethane-N,N,N′,N′-tetraacetic acid tetra(acetoxymethyl) ester.

C2C12 myotubes were treated with the fluorescent glucose analog 2-NBDG in the presence or absence of gintonin to measure the effect of gintonin on glucose uptake. Gintonin (3 μg/mL) increased glucose uptake up to 4 h in a time-dependent manner, and an incubation time of longer than 8 h did not further increase glucose uptake (Figure 2A). Gintonin at 0.3–30 μg/mL also dose-dependently induced glucose uptake (Figure 2B). Gintonin-stimulated glucose uptake was inhibited by treatment with the LPA1/3 receptor inhibitor Ki16425, the PLC inhibitor U73122, the intracellular calcium chelator 1,2-bis(o-aminophenoxy)ethane-N,N,N′,N′-tetraacetic acid tetra(acetoxymethyl) ester (BAPTA-AM), and the ERK inhibitor PD98059, indicating the involvement of LPA1/3 receptor activation, PLC activation, intracellular calcium increases, and ERK phosphorylation in the process (Figure 2C–F).

### 3.3. Effect of Gintonin on ATP and Glycogen Content of C2C12 Myotubes

**Figure 3:**
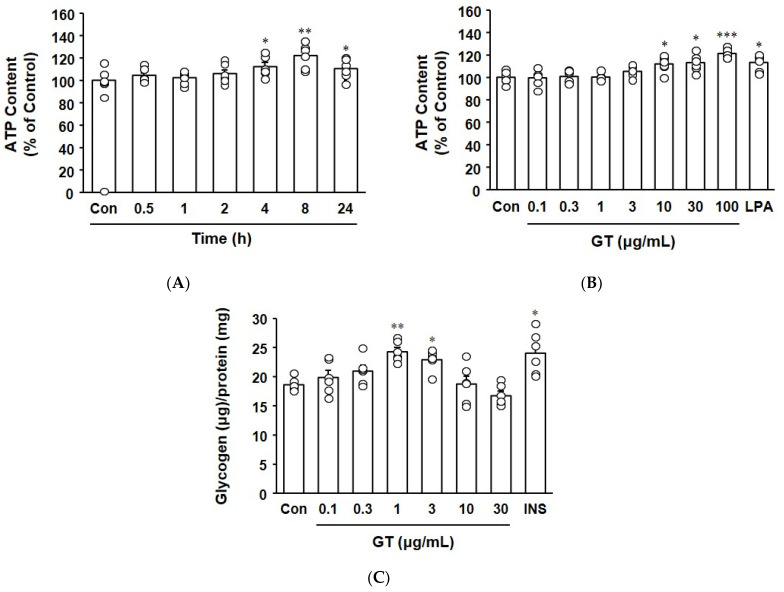
Effect of gintonin on ATP and glycogen content in C2C12 myotubes. (**A**,**B**) ATP content. (**A**) C2C12 myotubes were treated with gintonin (GT, 10 μg/mL) for 0–24 h. (**B**) The myotubes were treated with gintonin (0.1–100 μg/mL) or lysophosphatidic acid (LPA, 10 μM) for 8 h. The ATP content of myotubes was measured using an ATP assay kit. (**C**) Glycogen content. The myotubes were treated with gintonin (GT, 0.1–30 μg/mL) or insulin (INS, 100 nM), and the glycogen content was measured using a glycogen assay kit. All data are presented as the mean ± SEM (*n* = 6); * *p* < 0.05; ** *p* < 0.01; *** *p* < 0.001 vs. time 0 or untreated control cells (Con).

The effect of gintonin on the ATP content of myotubes was evaluated by treating C2C12 myotubes with gintonin and measuring cellular ATP content. As shown in Figure 3A, gintonin treatment for 4–24 h increased cellular ATP content, showing the highest content at 8 h of incubation. Gintonin treatment at 10–100 μg/mL increased the cellular ATP content (Figure 3B). These patterns are similar to the cell viability results in the WST-based assay (Figure 1A).

Cellular glycogen content was significantly increased by treatment with gintonin at 1 and 3 μg/mL. Gintonin (3 μg/mL) showed effects similar to those of insulin (100 nM), which was the positive control (Figure 3C).

### 3.4. Effect of Gintonin on ERK Phosphorylation in C2C12 Myotubes

**Figure 4:**
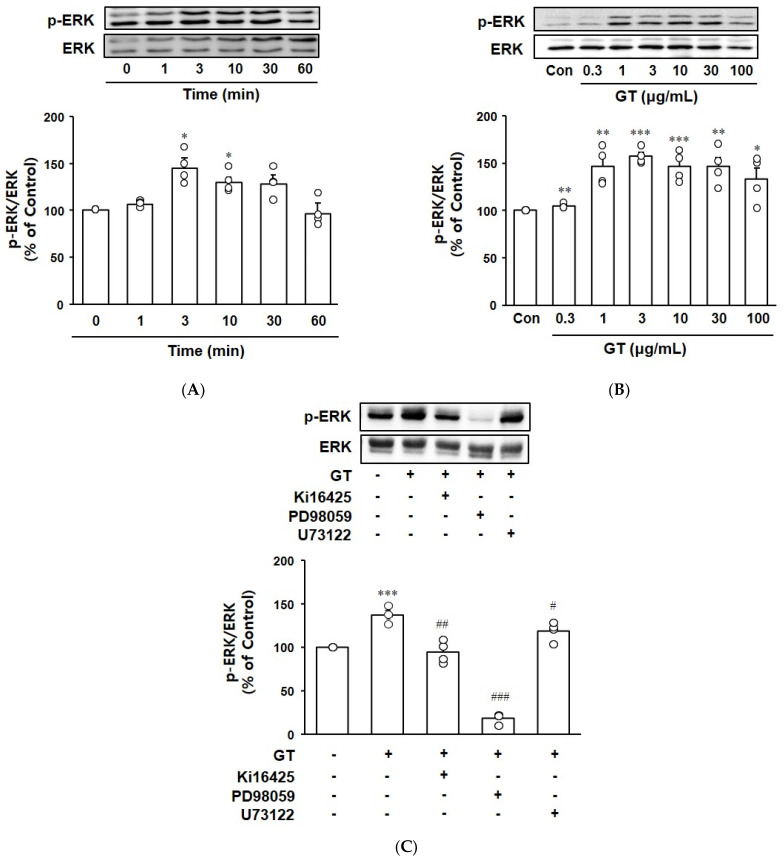
Effect of gintonin on ERK phosphorylation in C2C12 myotubes. (**A**) C2C12 myotubes were treated with gintonin (GT, 10 μg/mL) for 0–60 min. (**B**) The myotubes were treated with gintonin (0.3–100 μg/mL) for 10 min. (**C**) The myotubes were pretreated with inhibitors (Ki16425, 10 μM; PD98059, 10 μM; U73122, 5 μM) for 1 h and then treated with gintonin (3 μg/mL) for 10 min. Phosphorylated ERK and ERK were detected by immunoblotting. All data are shown as the mean ± SEM (n = 4); * *p* < 0.05; ** *p* < 0.01; *** *p* < 0.001 vs. time 0 or untreated control cells (Con). ^#^ *p* < 0.05; ^##^ *p* < 0.01; ^###^ *p* < 0.001 vs. GT alone. p-ERK, phospho-ERK. Original western blot images can be found in Supplementary File S1.

The effect of gintonin on ERK phosphorylation in the myotubes was assessed by treating C2C12 myotubes with gintonin and analyzing phospho-ERK and ERK expression by immunoblotting. Gintonin (10 μg/mL) induced ERK phosphorylation at 3 and 10 min of incubation (Figure 4A). Gintonin increased ERK phosphorylation at 0.3–100 μg/mL and reached a peak at 3 μg/mL (Figure 4B). Gintonin-induced ERK phosphorylation was inhibited by treatment with the LPA1/3 receptor inhibitor Ki16425, the PLC inhibitor U73122, and the ERK inhibitor PD98059, indicating the involvement of LPA1/3 receptor activation and PLC activation in ERK phosphorylation (Figure 4C).

### 3.5. Effect of Gintonin on GLUT4 Expression and Translocation in C2C12 Myotubes

**Figure 5:**
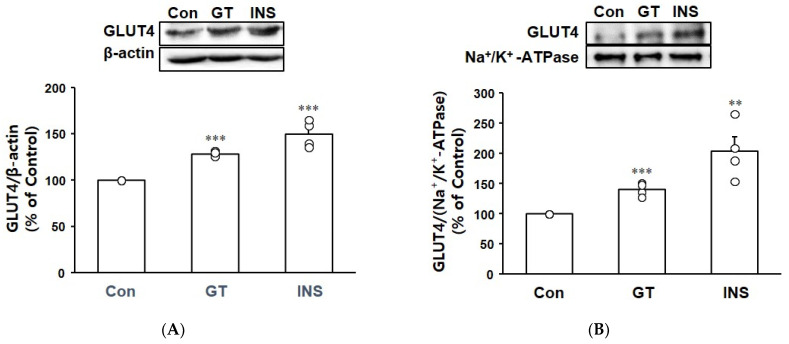
Effect of gintonin on GLUT4 expression in total lysates and plasma membrane fractions of C2C12 myotubes. (**A**) GLUT4 expression in total lysates. (**B**) GLUT4 expression in the plasma membrane fraction. C2C12 myotubes were treated with gintonin (GT, 10 μg/mL) for 120 min or insulin (INS, 100 nM) for 30 min. GLUT4 expression in total lysates and plasma membrane fraction of C2C12 myotubes was detected by immunoblotting. β-actin and Na^+^/K^+^ ATPase were also detected as loading controls. All data are shown as the mean ± SEM (*n* = 4); ** *p* < 0.01; *** *p* < 0.001 vs. untreated control cells (Con). Original western blot images can be found in Supplementary File S1.

GLUT4 plays a key role in glucose uptake. The effect of gintonin on the expression of GLUT4 in C2C12 myotubes was elucidated by treating the myotubes with gintonin and analyzing GLUT4 expression by immunoblotting. Gintonin increased the expression of GLUT4 in C2C12 myotubes (Figure 5A). GLUT4 expression was also measured in plasma membranes to observe the translocation of GLUT4 to the plasma membranes (Figure 5B). GLUT4 expression in the plasma membranes of C2C12 myotubes was increased by treatment with gintonin or insulin, indicating gintonin-enhanced GLUT4 translocation in the myotubes.

## 4. Discussion

In this study, we investigated the biological effects of gintonin on C2C12 myotubes, especially focusing on its influence on transient intracellular calcium increases, glucose uptake, ATP production, glycogen production, ERK phosphorylation, and GLUT4 expression and translocation. The results showed that gintonin exerted its beneficial effects on muscle metabolism-related responses, including glucose uptake.

**Figure 6:**
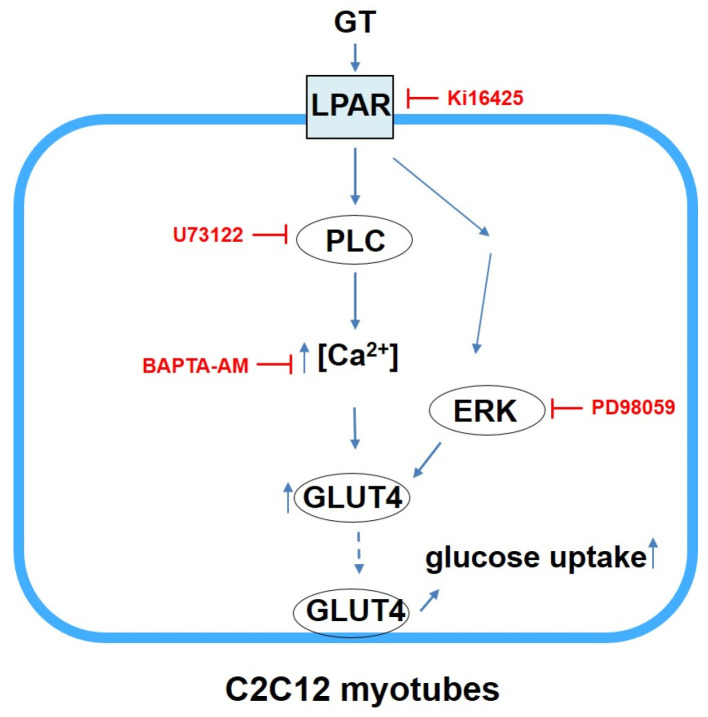
Possible signaling pathways of gintonin (GT)-induced glucose uptake in C2C12 myotubes. Gintonin induces transient increases in intracellular calcium concentrations and ERK activation via LPA receptor (LPAR) activation. These may lead to increases in the expression and translocation of GLUT4, subsequently increasing glucose uptake. PLC, phospholipase C; ERK, extracellular signal-regulated kinase; GLUT4, glucose transporter type 4.

Previous studies demonstrated that LPA receptor activation led to PLC-mediated IP_3_ signaling and subsequent calcium release [23,24,25,26,27]. Additionally, gintonin was reported to activate LPA receptors in different cell types, including B103 cells, vascular endothelial cells, astrocytes, and neuronal cells [15,16,28,29]. The present study showed that gintonin induced dose-dependent increases in intracellular calcium concentrations in C2C12 cells (Figure 1B,C). These increases by gintonin were inhibited by treatment with the LPA receptor inhibitor Ki16425, the PLC inhibitor U73122, and the IP_3_ receptor antagonist 2-APB (Figure 1D). Therefore, our findings suggest that gintonin may promote increases in intracellular calcium concentrations in C2C12 cells through LPA receptor activation, which subsequently triggers PLC-mediated IP_3_ signaling pathways (Figure 6).

Glucose uptake was reported to be involved in PLC activation and transient intracellular calcium increases in muscle cells [30,31,32]. Increased intracellular calcium can enhance glucose uptake by activating glucose transporters like GLUT4 [31,32]. Insulin stimulates glucose uptake through PLC/PI3K/AKT activation and stimulates MAP kinase ERK in muscle cells [33]. Intracellular calcium chelation reduced insulin-induced glucose transport in adipocytes and L6 myotubes, indicating the involvement of transient intracellular calcium increases in glucose transport [34,35]. In the present study, we observed that gintonin increased glucose uptake in C2C12 myotubes (Figure 2A,B). This increased glucose uptake was inhibited by the LPA1/3 receptor inhibitor Ki16425, the PLC inhibitor U73122, and the intracellular calcium chelator BAPTA-AM (Figure 2C–E). LPA receptors are expressed in C2C12 cells [36], and LPA stimulates glucose uptake in myotubes through LPA receptor activation [18]. Therefore, our results indicate that gintonin may have a potential role in glucose uptake through LPA receptor activation, PLC activation, and increases in intracellular calcium, leading to improvements in glucose metabolism in muscle.

Cellular energy metabolism in muscle cells can be linked to its effects on calcium signaling and glucose uptake [8,37,38]. Physiologically, glucose uptake in skeletal muscles is mainly activated by the insulin-dependent activation of PI3K/AKT [33], the activation of AMPK, and increases in intracellular calcium by exercise or some flavonoids, such as resveratrol [38,39]. Insulin stimulates glucose uptake and glycogen synthesis [33]. Muscle contraction involves transient intracellular calcium increases, glucose uptake, and ATP production [38]. In the present study, gintonin showed dual effects on glucose metabolism depending on its concentration. Gintonin treatment at 10–100 μg/mL induced a significant increase in ATP content in C2C12 myotubes, indicating that gintonin induced energy metabolism. In contrast, gintonin significantly increased glycogen content at concentrations of 1 and 3 μg/mL in C2C12 myotubes, suggesting a potential role for gintonin in promoting energy source storage in muscle cells. A gintonin-enriched fraction (GEF) protected against sarcopenic obesity by promoting energy use and reducing skeletal muscle atrophy in mice on a high-fat diet. Moreover, it also promoted the myogenic differentiation of skeletal muscle, showing increases in muscle mass and strength [20]. Gintonin also exhibited anti-atrophy effects in C2C12 myotubes and primary human normal skeletal myoblasts and protected against cancer cachexia in a mouse model [40]. It was suggested that those protective effects of gintonin may arise through LPA receptor activation [40]. Therefore, our findings indicate that gintonin may play a role in positively modulating muscle energy metabolism and energy storage, probably through calcium signaling, glucose uptake, glycogen synthesis, and ATP production. In this context, gintonin could be beneficial in conditions requiring increased muscle energy reserves.

ERK signaling is a critical pathway for regulating various cellular processes, including growth, migration, differentiation, and metabolism [41,42]. ERK activation participates in cellular growth and differentiation pathways critical for muscle function and adaptation [43]. In addition, activated ERK was shown to contribute to glucose uptake and glycogen storage [44,45,46]. LPA stimulates PI3K/AKT and ERK signaling through LPA receptor activation [47]. In the present study, we found that the LPA receptor ligand gintonin induced ERK phosphorylation in C2C12 myotubes (Figure 4). The LPA1/3 receptor inhibitor Ki16425 and the ERK inhibitor PD98059 inhibited gintonin-enhanced ERK phosphorylation, consistent with the glucose uptake results. Therefore, these findings suggest that gintonin might influence glucose metabolic outcomes and growth and differentiation in muscle cells via the ERK-mediated signaling pathway, as well as LPA receptor activation. Additionally, in the present study, PLC inhibitor only slightly inhibited gintonin-enhanced ERK phosphorylation, implying that other possible pathways by PLC activation may also exist for glucose uptake (Figure 6). Other potential pathways need to be examined in future studies.

Glucose uptake in skeletal muscle cells occurs through the glucose transporter GLUT4. GLUT4 expression and translocation were enhanced along with increased glucose uptake by muscle cells [33,38]. In the present study, gintonin enhanced the expression level of GLUT4 in both C2C12 myotubes and C2C12 myotube membranes (Figure 5). This indicates that gintonin may stimulate GLUT4 activity, such as promoting glucose uptake and facilitating the translocation of GLUT4 transporters to the cell surface. Although our study did not provide the detailed signaling pathways of GLUT4 expression and translocation, the findings suggest that gintonin may affect GLUT4 expression and translocation, probably leading to increases in glucose uptake. Therefore, gintonin has potential therapeutic implications for the management of metabolic disorders associated with impaired glucose homeostasis.

In summary, our findings demonstrated that gintonin exerts multifaceted biological effects on C2C12 myotubes through several processes, such as inducing intracellular calcium concentration increases via LPA receptor activation and IP_3_ signaling, enhancing glucose uptake and ATP and glycogen content, and activating the ERK signaling pathway. The results suggest that gintonin may modulate muscle cell metabolism and glucose uptake and that it may have the potential for use as a therapeutic agent for conditions related to muscle cell metabolism and function. Future research needs to further characterize the detailed effects of gintonin on GLUT4 dynamics and investigate the in vivo applications of gintonin for muscle metabolism and metabolic disorders associated with impaired glucose homeostasis.

## Data Availability

The data are contained within the article.

## Supplementary Materials

The following supporting information can be downloaded at: https://www.mdpi.com/article/10.3390/biom14101316/s1, File S1: original western blot images.
