# Supplementary material for: Gintonin Stimulates Glucose Uptake in Myocytes: Involvement of Calcium and Extracellular Signal-Regulated Kinase Signaling
Source: Biomolecules. 2024 Oct 17;14(10):1316. doi: 10.3390/biom14101316 (PMC11505745; doi:10.3390/biom14101316)
Supplement: Supplementary file 1 [file biomolecules-14-01316-s001.zip › Western band_original image_Lee et al_PR.pdf]

Original images of Western blots

Fig.4A

(a)

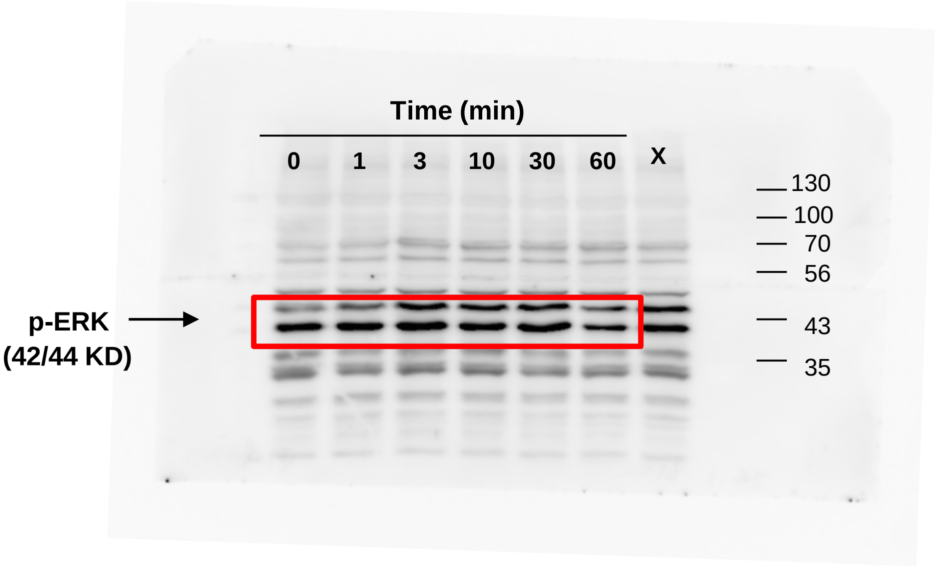

(b)

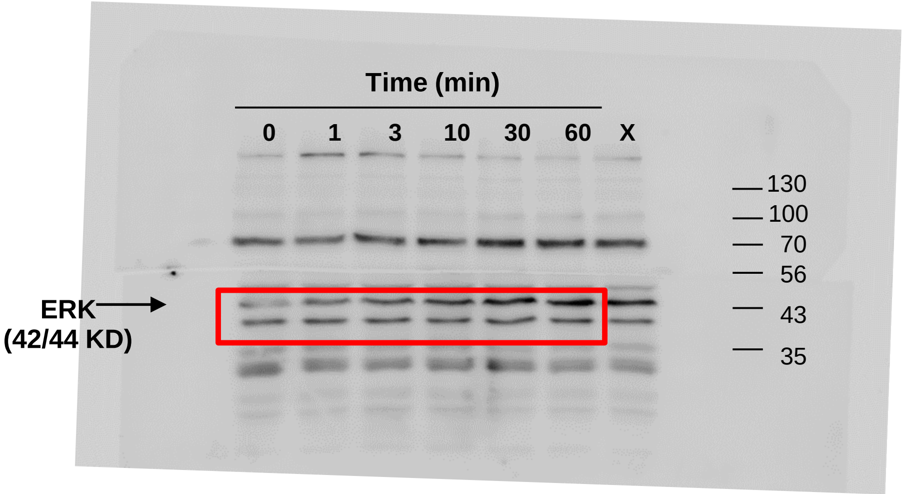

Membrane was cut at 56 KDa before incubation with primary antibodies. Lower part was used for detection of phospho-ERK (a) or ERK (b).

Fig.4B

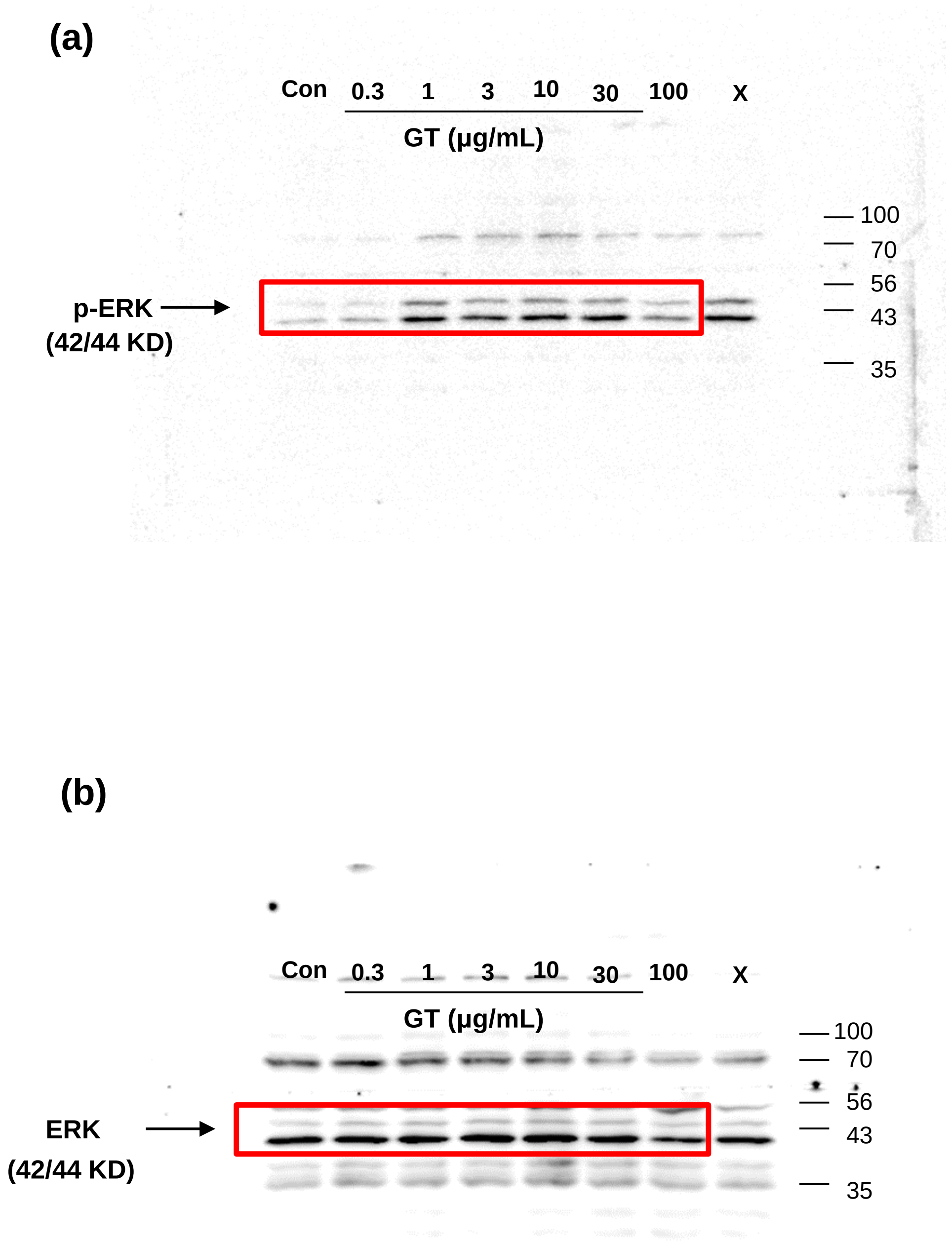

Membrane was cut at 56 KDa before incubation with primary antibodies.
Lower part was used for detection of phospho-ERK (a) or ERK (b).

Fig.4C

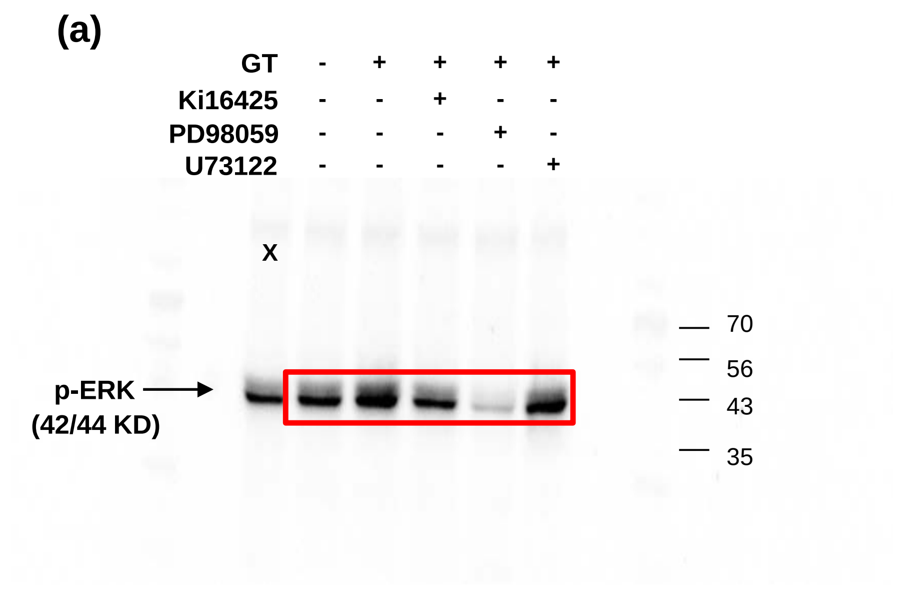

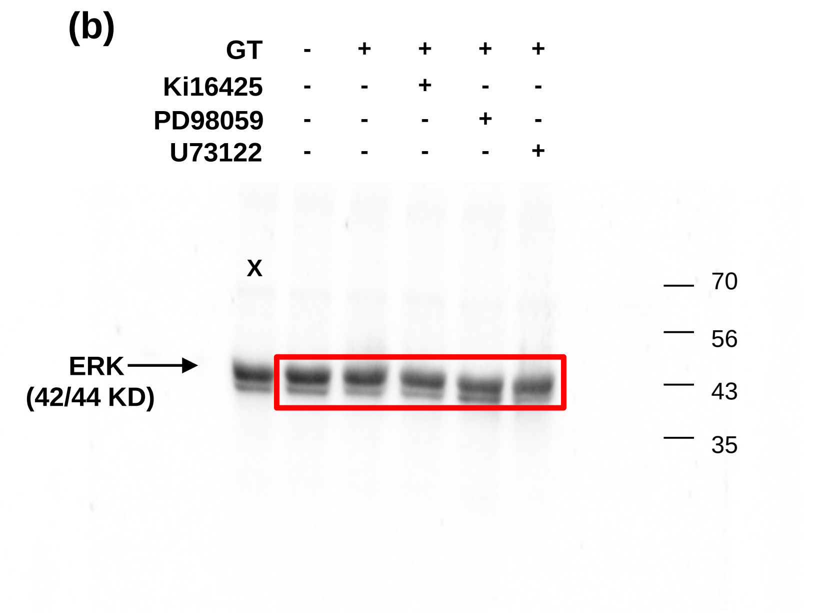

(a) Uncropped western blot image used for the cropped p-ERK image
(b) Uncropped western blot image used for the cropped ERK image

Fig.5A

(a)

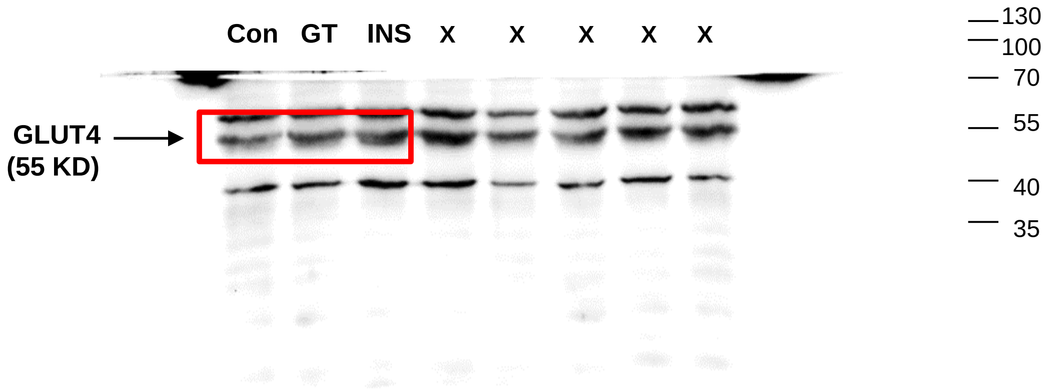

(b)

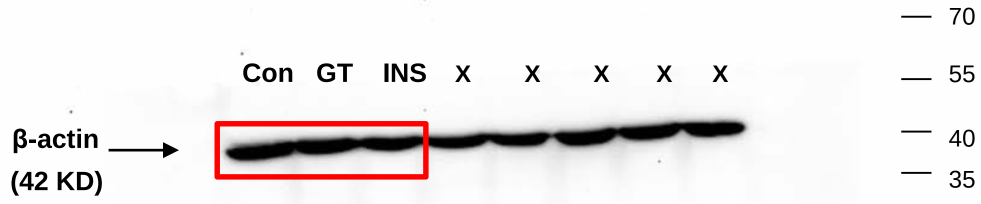

Membrane was cut at 70 KDa before incubation with primary antibodies.
Lower part was used for detection of GLUT4 (a) or β-actin (b).

Fig.5B

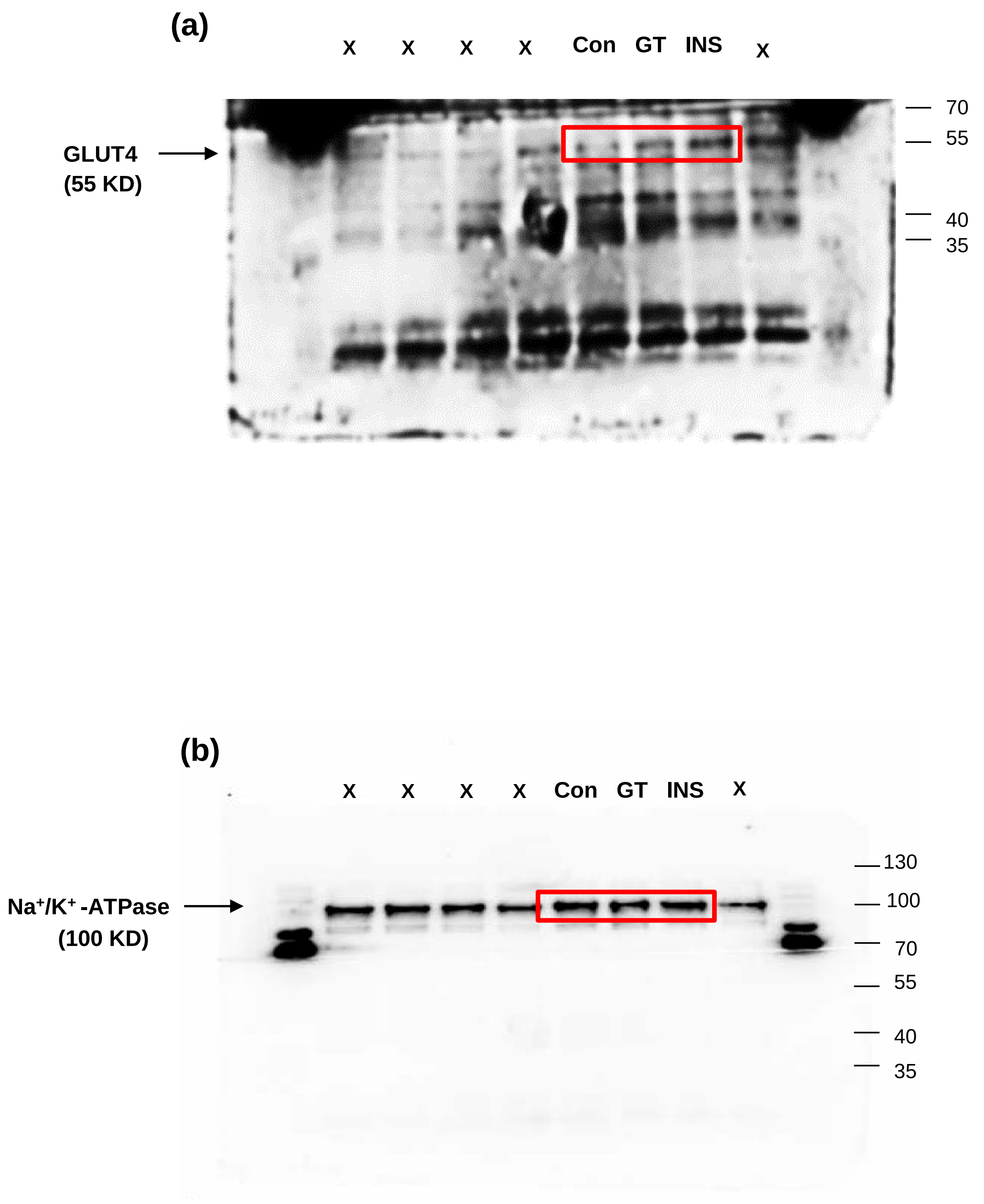

Membrane was cut at 70 KDa before incubation with primary antibodies.
Upper part was used for detection of Na^+/K^+ ATPase (b).
Lower part was used for detection of GLUT4 (a)
